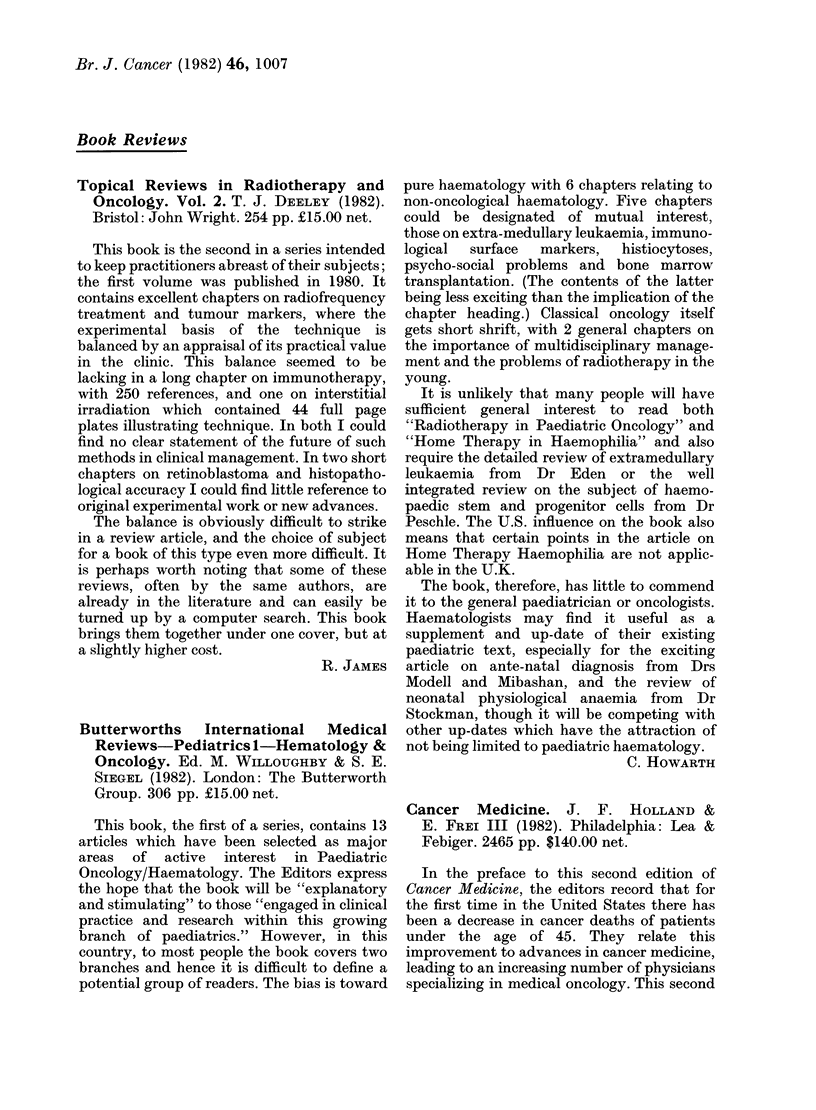# Butterworths International Medical Reviews—Pediatrics1—Hematology & Oncology

**Published:** 1982-12

**Authors:** C. Howarth


**Butterworths International Medical Reviews—Pediatrics 1—Hematology & Oncology.** Ed. M. WILLOUGHBY & S. E. SIEGEL (1982). London: The Butterworth Group. 306 pp. £15.00 net.

This book, the first of a series, contains 13 articles which have been selected as major areas of active interest in Paediatric Oncology/Haematology. The Editors express the hope that the book will be "explanatory and stimulating" to those "engaged in clinical practice and research within this growing branch of paediatrics." However, in this country, to most people the book covers two branches and hence it is difficult to define a potential group of readers. The bias is toward pure haematology with 6 chapters relating to non-oncological haematology. Five chapters could be designated of mutual interest, those on extra-medullary leukaemia, immunological surface markers, histiocytoses, psycho-social problems and bone marrow transplantation. (The contents of the latter being less exciting than the implication of the chapter heading.) Classical oncology itself gets short shrift, with 2 general chapters on the importance of multidisciplinary management and the problems of radiotherapy in the young.

It is unlikely that many people will have sufficient general interest to read both "Radiotherapy in Paediatric Oncology" and "Home Therapy in Haemophilia" and also require the detailed review of extramedullary leukaemia from Dr Eden or the well integrated review on the subject of haemopaedic stem and progenitor cells from Dr Peschle. The U.S. influence on the book also means that certain points in the article on Home Therapy Haemophilia are not applicable in the U.K.

The book, therefore, has little to commend it to the general paediatrician or oncologists. Haematologists may find it useful as a supplement and up-date of their existing paediatric text, especially for the exciting article on ante-natal diagnosis from Drs Modell and Mibashan, and the review of neonatal physiological anaemia from Dr Stockman, though it will be competing with other up-dates which have the attraction of not being limited to paediatric haematology.

C. HOWARTH